# Parity and bladder cancer risk: a dose-response meta-analysis

**DOI:** 10.1186/s12885-016-3023-5

**Published:** 2017-01-06

**Authors:** Yunjin Bai, Xiaoming Wang, Yubo Yang, Yin Tang, Jia Wang, Ping Han

**Affiliations:** Department of Urology, Institute of Urology, West China Hospital, Sichuan University, Guoxue Xiang#37, Chengdu, Sichuan 610041 China

**Keywords:** Parity, Bladder cancer, Meta-analysis, Women’s health

## Abstract

**Background:**

Multiple studies have reported evidence of an inverse association between parity and bladder cancer risk. However, a comprehensive and quantitative assessment of this association has never been conducted. We conducted this study to clarify this issue.

**Methods:**

Systematic search of PubMed and Embase was performed to identify all the studies. Studies were selected based on strict screening with inclusion and exclusion criteria. Summary relative risks (RR) with 95% confidence intervals (CI) were calculated by using a fixed-effect model, and the generalized least squares trend estimation was employed to compute study-specific RR and 95% CI per live birth increase. Heterogeneity and publication bias were also evaluated.

**Results:**

Twelve studies (6,214 cases and 2,693,350 non-cases) were eligible in this meta-analysis. The pooled RR of bladder cancer for parous versus nulliparous women was 0.76 (95% CI: 0.70–0.82). Results were similar in the studies that adjusted for BMI(RR = 0.66; 95% CI: 0.53–0.81), cigarette smoking (RR = 0.67; 95% CI: 0.57–0.79), and age (RR = 0.77; 95% CI: 0.71–0.84). The dose-response meta-analysis showed a lower bladder cancer risk (RR = 0.95; 95% CI: 0.92–0.98) for each live birth increase in parous women. No evidence of publication bias or significant heterogeneity was detected in the above-mentioned analyses.

**Conclusions:**

The finding from current meta-analysis suggest that parity may be related to decreased risk of bladder cancer.

**Electronic supplementary material:**

The online version of this article (doi:10.1186/s12885-016-3023-5) contains supplementary material, which is available to authorized users.

## Background

Bladder cancer (BC) is one of the most common genitourinary malignancies in the United States, with an estimated 76, 960 newly diagnosed cases and 16 390 deaths in 2016 [1]. Incidence rate of BC is notably gender-specific with men having a 3~4 fold greater risk than women for developing the disease [1]. Given the evidence from current knowledge, the gender disparity of incidence rate for BC cannot be completely explained by known risk factors, such as cigarette smoking and occupation [2].

Although the exact biological mechanisms for the gender disparity in BC development and progression are not well established, some studies have suggested that sex hormones and their receptors may contribute to this difference [3–5]. During pregnancy, changes in maternal hormones, as well as physical structure changes in the pelvic or lower abdominal region following childbirth, may lead to etiological changes that decrease BC risk. Till date, numerous observational studies [6–11] have investigated the roles of reproductive factors, such as age at menarche and menopause, parity, age at first birth, and number of children, in the development of BC, but results have been inconsistent, probably because of limited sample size included each individual study. Although published reviews and meta-analyses have focused on this topic [12, 13], up to now, a comprehensive and quantitative assessment of the relationship between parity number and BC risk has not been reported.

Therefore, we find it necessary to further assess the relationship between parity and BC risk by conducting a meta-analysis of current epidemiologic studies and providing a quantitative dose-response analysis.

## Methods

This study protocol was approved by the institutional review board at the West China School of Medicine before initiation and don’t need ethical standard statement.

## Literature search and eligibility criteria

We searched online databases MEDLINE and EMBASE for the studies assessing the relationship of parity and BC risk before Sep 2016, using the following terms with every possible combination considered: parity, pregnancy, reproductivity, fertility, bladder, genitourinary tract, cancer, carcinoma, tumor. We searched the references of all eligible publications to identify additional relevant publications. Studies were included if they met the following criteria: (1) the study had a prospective or case-control design; (2) the study evaluated the relationship between parity and BC risk; (3) data were provided or would allow the calculation of relative risk (RR) or odds ratio (OR) and a 95% confidence intervals (CI). Exclusion criteria were as follows: (1) was not involved with the associations between parity and BC risk; (2) non-human studies, reviews, and comments; (3) studies not reporting primary outcomes; (4) studies based on overlapping patients. When duplicate publications were identified or data in separate publications originated from the same cohort, we used the publication with the largest number of cases and the most applicable data.

### Data extraction and quality assessment

Data were independently extracted from the included studies by two investigators (YJB and XMW), and disagreement was settled by discussion with the third investigator. The following data were extracted from each study: first author’ name, publication year, country of study, participant demographics, sample size, duration of follow-up or study period, parity number categories, corresponding OR/RR (with their 95% CIs) for each category, and variables adjusted for in the analysis.

The methodological quality assessment was conducted for each of the included studies using the Newcastle-Ottawa Scale quality assessment [14] on three broad perspectives: selection, comparability and exposure or outcome. Two investigators read each included study and scored them independently. Disagreements were resolved by consensus.

### Statistical analysis

Given that bladder cancer is a rare disease, the OR was assumed to be the same as the RR and we therefore report all effect sizes as RR for simplicity. We calculated summary RRs and 95% CIs associated with parity and BC risk, if the study considered nulliparous as a reference. Between-study heterogeneity was evaluated with Q and I^2^ statistics [15]. For the Q-test, we used *P* < 0.10 as evidence of heterogeneity. An I^2^ score exceeding 50% is considered to indicate the presence of heterogeneity. The random effect model was used to provide summary estimations if there was heterogeneity, or else the fixed effect model was used. Heterogeneity was explored by stratified analysis. Publication bias was explored using Begg’s and Egger’s test [16, 17] and funnel plots. *P* < 0.05 indicated the existence of publication bias.

We carried out a dose-response meta-analysis using the method proposed by Greenland et al. [18] and Orsini et al. [19] to estimate study-specific slopes (linear trends) and 95% CIs from the log-RRs and CIs across the categories of parity number. This analysis requires the number of cases and controls for case-control studies or the number of cases and person-years for cohort studies, and the RR with 95% CI for at least three quantitative categories of parity number are presented. The value assigned to each category was the midpoint of the upper and lower boundaries and was adjusted for half range of the neighborhood categories when categories were open-ended. Random-effects model was applied in our study. This was done by modeling parity number using restricted cubic splines with three knots at percentiles 5, 50, and 95% of the distribution. A *P* value for nonlinearity was computed by testing the null hypothesis that the coefficient of the second spline was equal to zero. Statistical analyses were performed with Stata version 12.0 software (Stata Corp, College Station, TX).

## Results

### Study characteristics and quality assessment

**Fig. 1:**
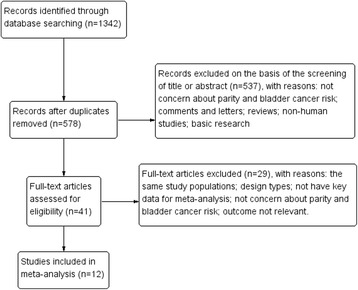
Flow diagram of the study selection process

The article selection process is shown in Fig. 1. Thirteen articles [6–13, 20–24] met the selection criteria. Two studies reported data on the same cohort [9, 22] and we included the more recently one which reported the largest number of cases [22]. Thus, 12 publications were included in this meta-analysis [6–8, 10–13, 20–24], and their characteristics are presented in Table 1. These studies included a total of 6,214 cases and 2,693,350 non-cases and were published from 1992 to 2013. Among these studies, one study reported three separate outcomes: the Los Angeles Bladder Cancer Study, the Shanghai Bladder Cancer Study, and the California Teachers Study [13]. Thus, the present study used seven outcomes from case-control studies and seven outcomes from cohort studies to produce a meta-analysis. Of the included studies, 13 were conducted in Europe, nine in America and one in China. All studies included met quality criteria ranging from 5 to 7 stars.

**Table 1:** Characteristics of eligible studies included in this meta-analysis of parity and bladder cancer risk

Author, year	Design, study name	Country, study period	Study quality^a^	Age	Cases; participants/controls	Parity	OR (95% CI)	Adjustment factors
Cantor et al. 1992 [20]	Case-control study	USA, 1986–1989	5	40–85	315; 821	1	Reference	Not reported
						2	1.4 (0.8–2.4)	
						3	1.6 (0.9–2.9)	
						4	0.9 (0.5–1.8)	
						5+	1.5 (0.8–2.7)	
La Vecchia et al. 1993 [21]	Case-control study	Italy, 1983–1992	6	Case: blow 70; Control: 58 (median)	68; 5619	0	Reference	Age, education, parity, number of abortions, oral contraceptive and other female hormone use
						1	1.2	
						2	1.0	
						3	0.8	
						≥4	0.7	
Pelucchi et al. 2002 [10]	Case-control study	Italy, 1985–1992	6	Case: 30–79 Control: 26–79	110; 289	0–1	Reference	Age, study center, education, BMI, cigarette smoking, and coffee and alcohol consumption
						2–3	0.83 (0.47–1.45)	
						≥4	0.88(0.41–1.90)	
Cantwell et al. 2006 [8]	Cohort study	USA, 1980–1998	7	Case: 55.4 ± 8.8 Participants: 70.6 ± 8.4,	167; 54308	0	Reference	Age, calendar year and smoking status (never, former or current).
						1	0.65 (0.36–1.18)	
						2	0.96 (0,61–1,。50)	
						3	0.82 (0.5–1.34)	
						>4	0.75 (0.45–1.25)	
McGrath et al. 2006 [11]	Cohort study, US Nurses’ Health Study	USA, 1976–2002	7	30–55	336; 116598	0	Reference	Age (months), time period (2-year questionnaire period), smoking status (never, former, current) and pack-years of smoking (continuous), and body mass index
						1–2	0.85 (0.56–1.28)	
						3–4	0.70 (0.47–1.05)	
						≥4	0.84 (0.54–1.32)	
Prizment et al. 2006 [22]	Cohort study, The Iowa Women’s Health Study	USA, 1986–2003	7	55–69	192; 37459	0	Reference	Age, smoking status and pack-years
						1–2	0.81 (0.50–1.32)	
						3–4	0.79 (0.48–1.27)	
						≥5	0.79 (0.46–1.37)	
Huang et al. 2009 [7]	Case-control study	Spanish, 1998–2001	6	Median: case 67.4	152; 166	0	Reference	Age, smoking status, and high-risk occupation
				Control:67.8		≥1	0.43 (0.2–0.9)	
Davis-Dao et al. 2011 [13]		USA and China,	6	LAS:25–64;	LAS:349/349	LA:1	LA: Reference	LASBCS: smoking status in the reference year (current, former, never), pack-years of smoking, and BMI; CTS: race/ethnicity, smoking status and BMI.
	Los Angeles Bladder Cancer Study (LAS): Case-control study;	Los Angeles: 1987–1996;		SHS:25–74;	SHS: 131/138	2	0.41 (0.98–0.87)	
				CTS: not reported	CTS: 196/120857	3	0.63 (0.29–1.34)	
						≥4	0.70 (0.35–1.39)	
	Shanghai Bladder Cancer Study (SHS): Case-control study	Shanghai:1995–1998;				SH: 1	SH: reference	
						2	0.63 (0.23–1.74)	
						3	0.47 (0.16–1.33)	
	California Teachers Study(CTS): Cohort study	CTS:1995–2005				≥4	0.56 (0.21–1.48)	
						CTS:1	CTS: reference	
						2	1.14 (0.71–1.86)	
						3	0.73 (0.42–1.27)	
						≥4	1.06 (0.61–1.87)	
Dietrich et al. 2011 [12]	Case-control study	USA, 1994–2001; 1993	6	25–74	207/403	0	Reference	Age and smoking status
		July; 1995–1997				1–2	0.67 (0.36–1.24)	
						3–4	0.75 (0.40–1.39)	
						≥5	0.74 (0.35–1.57)	
Daugherty et al. 2013 [23]	Cohort study, NIH-AARP Diet and Health Study	USA, 1995–2006	7	50–71	651/201492	0	Reference	Age
						1	0.78 (0.58–1.07)	
						2	0.72 (0.56–0.92)	
						3–4	0.78 (0.62–0.98)	
						5+	0.73 (0.54–0.98)	
Kabat et al. 2013 [24]	Cohort study, Women’s Health Initiative	USA, 1993–1998	6	50–79	480/145048	0	Reference	Age, education, pack-years, alcohol intake, parity, oral contraceptive use (ever, never)
						1–2	0.71 (0.53–0.96)	
						3–4	0.84 (0.63–1.12)	
						≥5	0.72 (0.51–1.03)	
Weibull et al. 2013 [6]	Cohort study	Sweden, 1964–2009, Median follow-up time:16.6 years	7	40~	2860/2009811	1	Reference	Age, highest educational level, and chronic obstructive lung disease.
						2	0.85 (0.77–0.95)	
						≥3	0.76 (0.68–0.86)	

^a^Using the Newcastle-Ottawa Scale

### Ever versus never parity

**Fig. 2:**
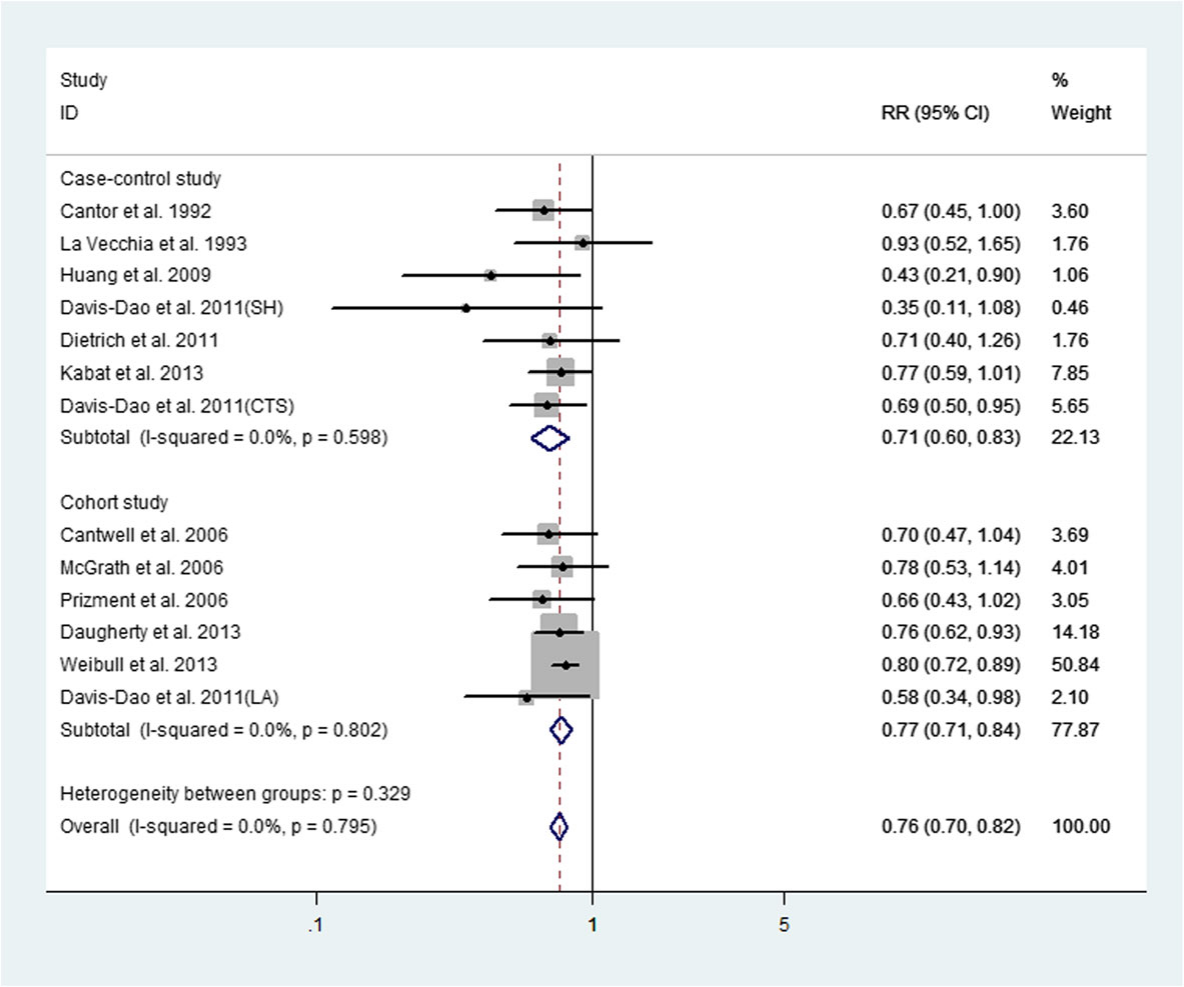
Forest plot (fixed-effects model) of ever parity and bladder cancer risk

All included studies in present study investigated the association between ever parity and BC risk. The summary multivariable-adjusted RR of BC for ever versus nulliparous was 0.76 (95% CI, 0.70–0.82), without heterogeneity (I^2^ = 0%, *p* = 0.795, Fig. 2), indicating an inverse association between parity and BC risk. For women who have never smoked, the summary RR of BC for ever versus nulliparous was 0.47 (95% CI, 0.35–0.63), without heterogeneity (I^2^ = 0%, *p* = 0.562). For women who have ever smoked, the summary RR was 0.90 (95% CI, 0.67–1.21) for ever versus nulliparous, without heterogeneity (I^2^ = 0%, *p* = 0.531).

### Differences with parity number

Six cohort and six case-control studies investigated possible association between parity and BC risk. The results of the effects of different parity number on BC risk are presented in Table 2. To clarify the effects, we divided the parity number into three groups. The first group (1–2 births) contained seven reports, compared with nulliparous,, the pooled RR of BC associated with giving birth to two children was 0.82 (95% CI 0.71–0.94) with I^2^ = 17.7% (*P* = 0.295). The second group (3–4 births) contained seven reports, compared with nulliparous, the overall RR of BC was 0.79 (95% CI 0.68–0.91) with I^2^ = 0% (*P* = 0.997). The third group (≥5 births) contained six reports, compared with nulliparous, the overall RR of BC was 0.76 (95% CI 0.66–0.88) with I^2^ = 0% (*P* = 0.994).

**Table 2:** Summary risk estimates of the association between parity and bladder cancer

	No. of reports	RR (95% CI)	I^2^ (%)	*P* value
Overall	13	0.76 (0.70–0.82)	0	0.795
Number of parity				
1 ~ 2 vs. 0	7	0.82 (0.71–0.94)	17.7	0.295
3 ~ 4 vs. 0	7	0.79 (0.68–0.91)	0	0.997
≥ 5 vs. 0	6	0.76 (0.66–0.88)	0	0.994
Subgroup analysis				
Study design				
Cohort study	6	0.77 (0.71–0.84)	0	0.802
Case-control study	7	0.71 (0.60–0.83)	0	0.598
Number of cases				
< 250	7	0.68 (0.56–0.81)	0	0.673
> 250	6	0.78 (0.71–0.84)	0	0.841
Location				
USA	9	0.72 (0.65–0.81)	0	0.989
Others	4	0.79 (0.71–0.87)	39.7	0.174
Smoking				
Never smoking	5	0.47 (0.35–0.63)	0	0.562
Ever smoking	4	0.90 (0.67–1.21)	0	0.531
Adjustment for smoking				
Yes	8	0.67 (0.57–0.79)	0	0.815
No	5	0.79 (0.72–0.86)	0	0.882
Adjustment for age				
Yes	9	0.77 (0.71–0.84)	0	0.850
No	4	0.65 (0.52–0.81)	0	0.684
Adjustment for BMI				
Yes	5	0.66 (0.53–0.81)	0	0.469
No	8	0.76 (0.70–0.82)	0	0.952

### Dose-response meta-analysis

**Fig. 3:**
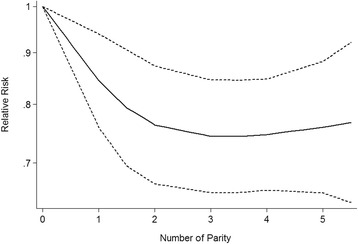
Nonlinear dose-response relationship between parity and bladder cancer risk

Dose-response from nine studies [6, 8, 11–13, 20–23] showed a decreased in BC risk of 0.95 (95% CI: 0.92–0.98) per live birth increase, without heterogeneity (I^2^ = 12.79%, *P* = 0.7832). Compared with nulliparous individuals, the pooled RRs (95% CI) of BC were 0.84 (0.76–0.94), 0.76 (0.67–0.87), 0.74 (0.65–0.85), 0.75 (0.66–0.85), and 0.76 (0.65–0.88) for 1, 2, 3, 4, and 5 live births, respectively (Fig. 3).

### Publication bias

No evidence of a significant publication bias was observed in our analyses as assessed using the Egger’s test or Begg’s test and no asymmetry was seen in the funnel plots of included studies.

### Subgroup analyses

To avoid the influence of cigarette smoking, we evaluated the influence of smoking status by adjustment in the eight reports that considered smoking. However, when data were stratified by adjustment for smoking, we did not find a significant difference between summary RRs that were adjusted and those that were not adjusted for smoking (Table 2). Moreover, significant inverse associations were persisted after the subgroup analyses stratified by whether the study included adjustment for specific potential confounders, such as age, Body Mass Index (BMI), type of control subjects or others potential confounders (Table 2). An additional file shows this in more detail (see Additional file 1).

## Discussion

Numerous epidemiological studies have focused on BC risk factors in order to explain the gender disparity in BC incidence, while there is also a great interest in identifying factors that affect the risk of BC among women. Although previous studies have reported on the associations between parity and BC, to the best of our knowledge, no other earlier studies have clearly documented a dose-response pattern between parity number and BC risk. In present study, we conducted a dose-response meta-analysis to characterize the association between parity and BC risk. Findings from the present study indicated that parous was significantly inversely associated with BC risk compared with nulliparous, especially for non-smokers. Overall, the risk of BC decreased by 24% for women who had given birth and 53% for those who had never smoked and were ever parous.

Although the exact mechanisms underlying this inverse association between parity and BC risk are not completely established, several potential mechanisms have been proposed. First, during pregnancy, estrogen and progesterone levels increase drastically and induce substantial alterations to the structure, function, and histology of the bladder [25], possibly affecting the development and progression of BC. Both estrogen and progesterone receptors are found in bladder tissues suggesting that endocrine regulation could directly influence BC development [26]. Shen et al. [27] found that antiestrogens have an inhibitory effect on the growth of BC cells in vitro, indicating that estrogens may increase the development and growth of bladder malignancies. Some research has reported that progesterone suppresses the activity of the estrogen receptor during pregnancy [28]. Therefore, estrogen and progesterone have an antagonistic effects on human cells. The substantial increased in their levels during pregnancy may responsible for the decreased risk of BC for women. Moreover, a hormone-related protective effect based on the prity number may mirror the lifetime period of lactation and prolonged oxytocin inhibition of steroid hormones [6].

Second, smoking habits may have influenced the incidence of BC in women with parity. It is well known that prenatal cigarette smoking exposure may cause detrimental effects on reproductive health [29]. Therefore, most smokers who prepare for pregnancy are more likely to quit smoking. Thus, compared with nulliparous female smokers, parous smokers who quit smoking may have a shorter smoking exposure history and reduction of pack years, and this may affect their BC risk. In addition, cumulative effect of abandon smoking following multiple pregnancies may also associate to decrease the subsequent risk of BC. The findings of our study suggest that the negative correlation between parity and BC appears more remarkable among women who were never exposed to cigarettes, with these parous women experiencing at least a 53% decreased risk of BC compared with nulliparous women. However, such a dramatic risk reduction was not apparent among smokers. The reasons for this results of subgroup analysis may be that the effect of smoking on BC is so huge that for the development of the disease minor influence factors such as parity are less relevant. In case of never smokers, these minor influence factors play indeed a role.

Third, structural changes in the pelvic organs and pelvic floor organization following pregnancy may induce a decreased risk for BC in parous women. Childbirth is an established risk factor for lower urinary tract dysfunction, such as increased urinary frequency, stress incontinence, and overactive bladder [30], but the possible relationship with BC remains unclear. It is well known that BC risk is related to the contact time of the urothelium with carcinogens in urine. Some study reported that increased water intake and urination frequency may lessen contact time of urinary carcinogens with urothelium of the bladder, thus, diminish the bladder cancer risk [31, 32]. Zhang et al. [31] found that increasing urination frequency during daytime was associated with decreased risk of bladder cancer. Silverman et al. [32] suggested that nocturia may be a powerful factor in reducing BC risk. Based on the results of above studies, we can be reasonable to assume that lower urinary tract dysfunction after childbirth has a protective effect on BC risk, and this may be associated with shorter duration of exposure to urine carcinogens in the bladder. Further research should verify this hypothesis.

Meta-analyses of observational studies are prone to confounding, selection, and information bias as the included observational studies and can present particular challenges that may distort the results. However, our study has some strengths. First, we searched 2 electronic databases, which ensured a broad scope, and we included seven cohorts and seven case-control studies that involved a total of 6,214 cases and 2,693,350 non-cases. This large sample size should have provided sufficient statistical power to detect this potential relationship. Second, to control misclassification, we conducted a dose-response meta-analysis. This is the most comprehensive meta-analysis evaluating the associations between parity and BC. Third, no publication biases were detected in present study and outcome may be unlikely to remain unreported as studies with complex assessment of exposure.

## Conclusions

This current dose-response meta-analysis indicates that women with parity have an inversely associated BC risk compared with nulliparous women. The exact mechanism underlying this protective effect requires further investigation.

## Additional file

Additional file 1: Subgroup analyses of forest plots included in our meta-analysis. (ZIP 143 kb)

## Abbreviations

BC: Bladder cancer
BMI: Body mass index
CI: Confidence interval
OR: Odds ratio
RR: Relative risk
