# Supplementary figures and images for: Parity and bladder cancer risk: a dose-response meta-analysis
Source: BMC Cancer. 2017 Jan 6;17:31. doi: 10.1186/s12885-016-3023-5 (PMC5219774; doi:10.1186/s12885-016-3023-5)

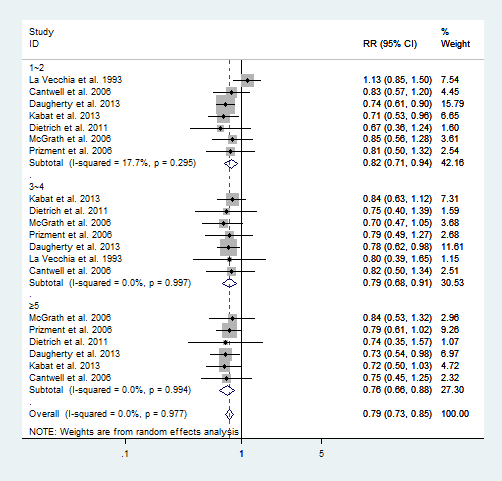

Supplement: Additional file 1: — Subgroup analyses of forest plots included in our meta-analysis. (ZIP 143 kb) [file 12885_2016_3023_MOESM1_ESM.zip › Figure S1. Differences with parity numberR3.tif]

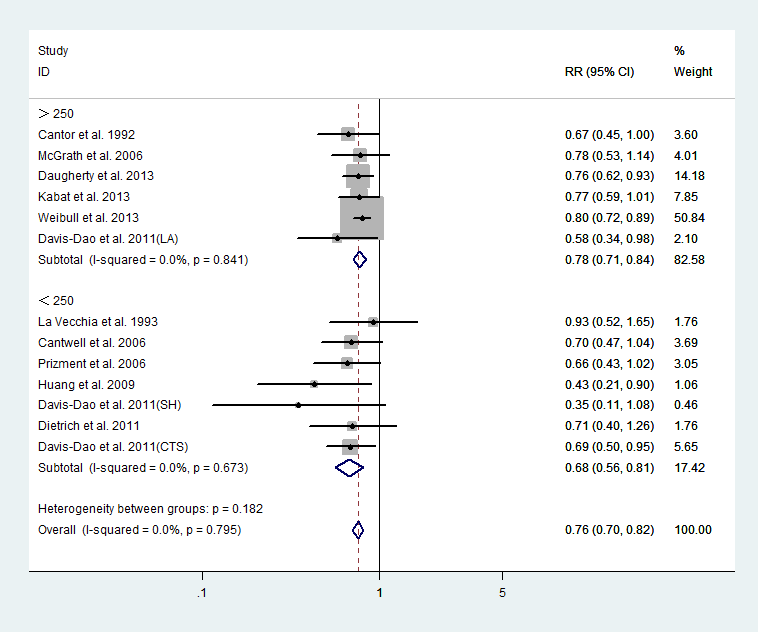

Supplement: Additional file 1: — Subgroup analyses of forest plots included in our meta-analysis. (ZIP 143 kb) [file 12885_2016_3023_MOESM1_ESM.zip › Figure S2. sample size of include studyR3.tif]

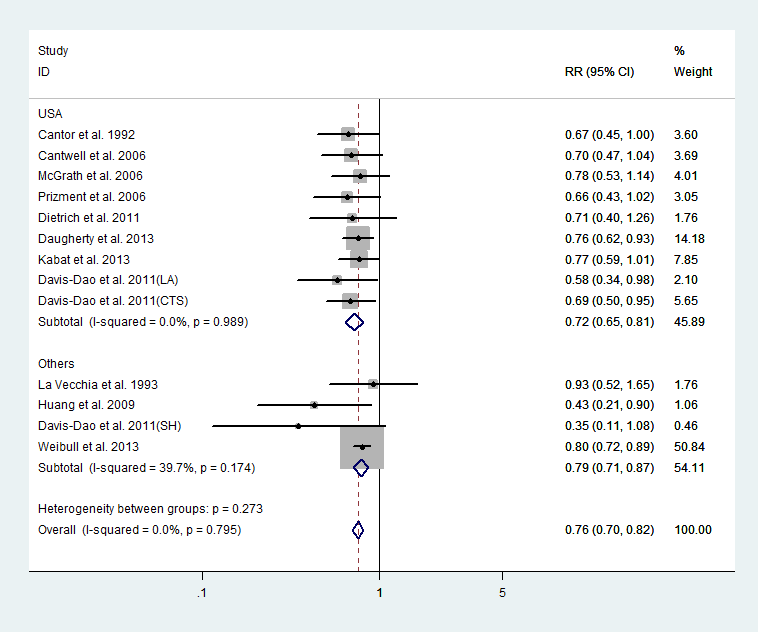

Supplement: Additional file 1: — Subgroup analyses of forest plots included in our meta-analysis. (ZIP 143 kb) [file 12885_2016_3023_MOESM1_ESM.zip › Figure S3. locationR3.tif]

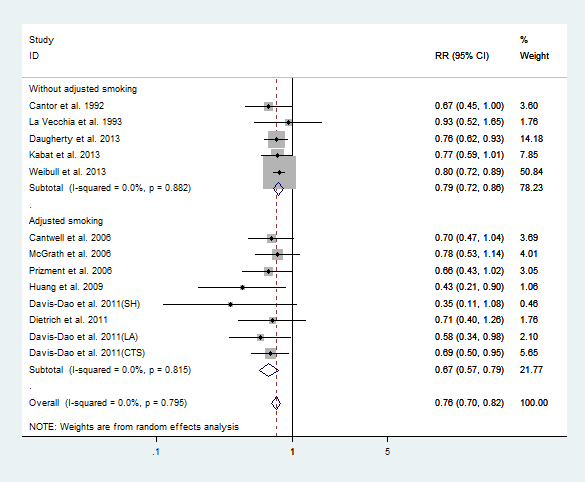

Supplement: Additional file 1: — Subgroup analyses of forest plots included in our meta-analysis. (ZIP 143 kb) [file 12885_2016_3023_MOESM1_ESM.zip › Figure S4. adjustment for smokingR3.tif]

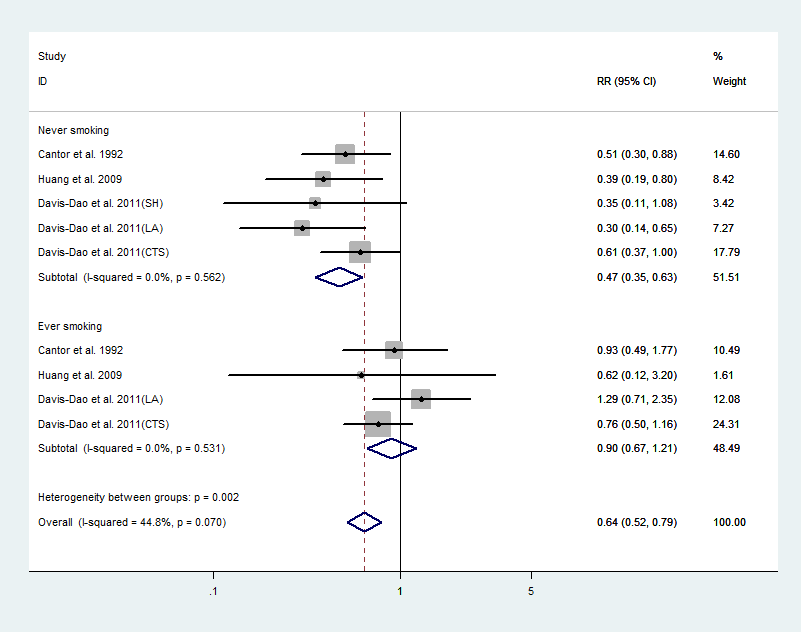

Supplement: Additional file 1: — Subgroup analyses of forest plots included in our meta-analysis. (ZIP 143 kb) [file 12885_2016_3023_MOESM1_ESM.zip › Figure S5. never vs. ever smokingR3.tif]

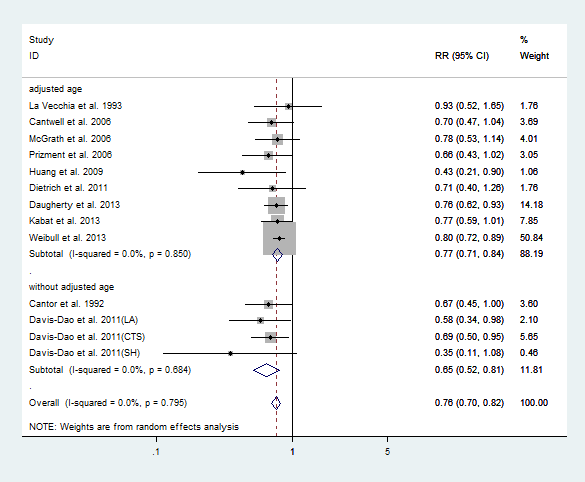

Supplement: Additional file 1: — Subgroup analyses of forest plots included in our meta-analysis. (ZIP 143 kb) [file 12885_2016_3023_MOESM1_ESM.zip › Figure S6. adjusted ageR3.tif]

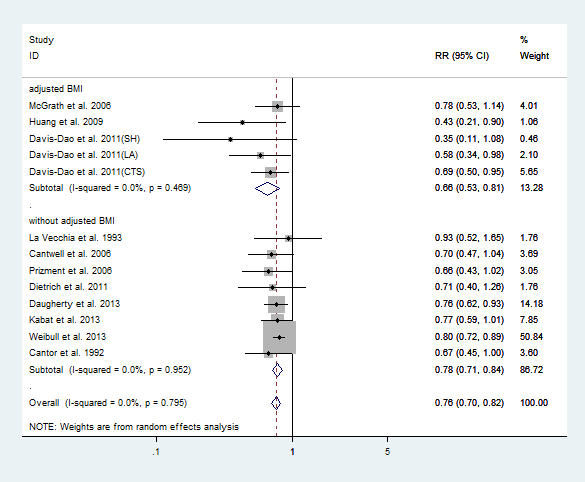

Supplement: Additional file 1: — Subgroup analyses of forest plots included in our meta-analysis. (ZIP 143 kb) [file 12885_2016_3023_MOESM1_ESM.zip › Figure S7. adjusted BMIR3.tif]
